# Supplementary figures and images for: The impact of longstanding illness and common mental disorder on competing employment exits routes in older working age: A longitudinal data-linkage study in Sweden
Source: PLoS One. 2020 Feb 25;15(2):e0229221. doi: 10.1371/journal.pone.0229221 (PMC7041791; doi:10.1371/journal.pone.0229221)

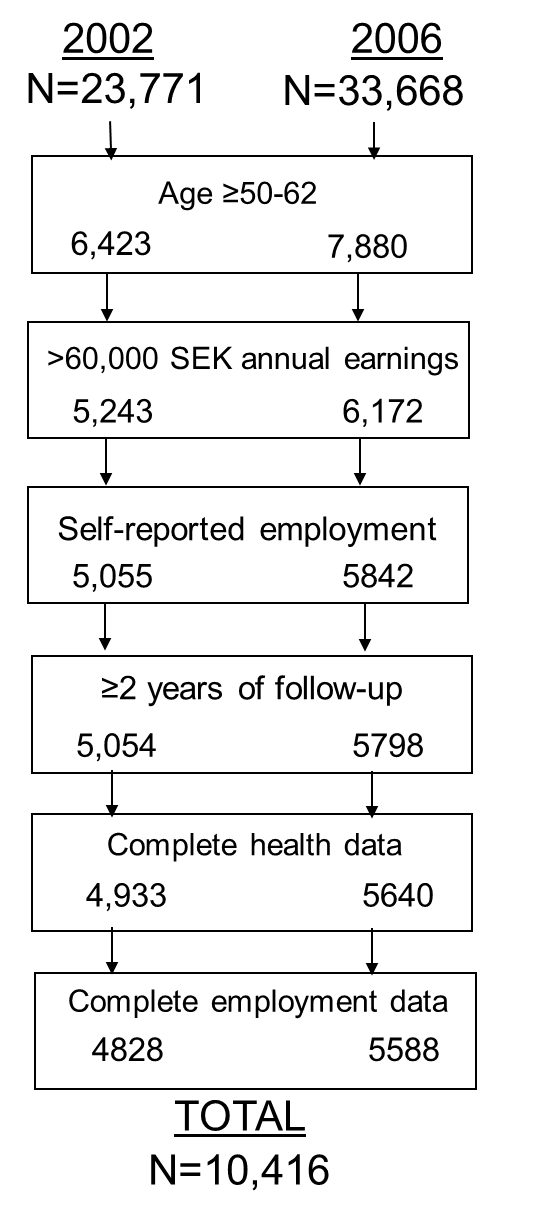


**Figure S1 Process of deriving the analytical sample**

Supplement: S1 Fig — (DOCX) [file pone.0229221.s001.docx]
