# Supplementary material for: The impact of longstanding illness and common mental disorder on competing employment exits routes in older working age: A longitudinal data-linkage study in Sweden
Source: PLoS One. 2020 Feb 25;15(2):e0229221. doi: 10.1371/journal.pone.0229221 (PMC7041791; doi:10.1371/journal.pone.0229221)
Supplement: S1 Table — (DOCX) [file pone.0229221.s002.docx]

| **Table S1. Unadjusted competing risks analyses on the influence of health, demographic, and socio-economic factors and work conditions at baseline among employed persons on the likelihood of employment exit (N = 10,416)** | | | | | | | | | |
| --- | --- | --- | --- | --- | --- | --- | --- | --- | --- |
|  | **Early retirement**  **(n=3783/n=10,416)** | | | **Disability pension (n=520/n=10,416)** | | | **Unemployment**  **(n=718/n=10,416)** | | |
|  | SHR | (95% CI) | *p* | SHR | (95% CI) | *p* | SHR | (95% CI) | *p* |
| **Comorbidity** |  |  |  |  |  |  |  |  |  |
| No LLI, No CMD | 1.00 |  |  | 1.00 |  |  | 1.00 |  |  |
| LLI only | 0.89 | (0.75-1.04) | 0.147 | 12.99 | (10.67-15.82) | <0.001 | 1.10 | (0.91-1.34) | 0.316 |
| CMD only | 0.93 | (0.73-1.19) | 0.565 | 1.93 | (1.27-2.92) | 0.002 | 1.67 | (1.33-2.08) | <0.001 |
| LLI and CMD | 0.96 | (0.69-1.32) | 0.791 | 17.00 | (13.05-22.14) | <0.001 | 1.08 | (0.75-1.55) | 0.678 |
| **Women** | 0.98 | (0.87-1.09) | 0.679 | 2.04 | (1.71-2.43) | <0.001 | 0.99 | (0.86-1.13) | 0.827 |
| **Age** | 1.19 | (1.17-1.22) | <0.001 | 1.72 | (1.62-1.83) | <0.001 | 1.27 | (1.23-1.32) | <0.001 |
| **Born outside Sweden** | 0.68 | (0.56-0.82) | <0.001 | 1.23 | (0.99-1.52) | 0.059 | 1.46 | (1.23-1.73) | <0.001 |
| **Single, divorced, widowed** | 0.68 | (0.60-0.78) | <0.001 | 1.14 | (0.97-1.34) | 0.119 | 1.29 | (1.13-1.48) | <0.001 |
| **Education** |  |  |  |  |  |  |  |  |  |
| Primary | 1.15 | (0.97-1.36) | 0.113 | 1.63 | (1.29-2.05) | <0.001 | 1.77 | (1.44-2.16) | <0.001 |
| Secondary | 1.23 | (1-09-1.40) | 0.001 | 1.24 | (1.04-1.48) | 0.018 | 1.54 | (1.33-1.80) | <0.001 |
| University | 1.00 |  |  | 1.00 |  |  | 1.00 |  |  |
| **Social occupational class** |  |  |  |  |  |  |  |  |  |
| High non-manual | 1.00 |  |  | 1.00 |  |  | 1.00 |  |  |
| Intermediate non-manual | 1.14 | (0.99-1.31) | 0.073 | 1.48 | (1.18-1.86) | 0.001 | 1.38 | (1.13-1.68) | 0.002 |
| Low non-manual | 0.96 | (0.82-1.12) | 0.559 | 2.01 | (1.62-2.50) | <0.001 | 2.18 | (1.81-2.62) | <0.001 |
| Manual | 0.77 | (0.63-0.93) | 0.007 | 1.74 | (1.35-2.23) | <0.001 | 2.09 | (1.70-2.57) | <0.001 |
| **Financial strain** | 0.63 | (0.45-0.87) | 0.006 | 1.59 | (1.20-2.09) | 0.001 | 1.56 | (1.23-1.97) | <0.001 |
| **Employment conditions** |  |  |  |  |  |  |  |  |  |
| Employed, great work freedom | 1.00 |  |  | 1.00 |  |  | 1.00 |  |  |
| Employed, limited work freedom | 1.26 | (1.06-1.49) | 0.009 | 2.10 | (1.71-2.57) | <0.001 | 1.59 | (1.32-1.91) | <0.001 |
| Self-employed | 1.13 | (1.08-1.53) | 0.004 | 0.86 | (0.65-1.16) | 0.326 | 0.75 | (0.58-0.96) | 0.023 |
| SHR: Subdistribution hazard ratio. | | | | | | | | | |
